# Intracavitary Drainage Followed by Lobectomy for a Giant Bulla Causing Respiratory Failure due to Severe Mediastinal Compression: A Case Report

**DOI:** 10.70352/scrj.cr.26-0135

**Published:** 2026-06-02

**Authors:** Hikaru Tokutake, Hironobu Wada, Yuki Onozato, Toshiko Kamata, Takashi Anayama, Ichiro Yoshino, Shigetoshi Yoshida

**Affiliations:** 1 Department of Thoracic Surgery, International University of Health and Welfare Narita Hospital, Narita, Chiba, Japan; 2 Department of Thoracic Surgery, International University of Health and Welfare School of Medicine, Narita, Chiba, Japan

**Keywords:** giant emphysematous bullae, intracavitary drainage, volume reduction surgery

## Abstract

**INTRODUCTION:**

We present the case of a giant emphysematous bulla resulting in deteriorating respiratory condition due to severe mediastinal compression that was successfully treated with emergency intracavitary drainage followed by elective right upper lobectomy.

**CASE PRESENTATION:**

A 57-year-old man was admitted to our hospital because he developed progressive dyspnea over 1 month and was eventually unable to ambulate. His oxygen saturation was 89% in room air. A chest radiograph showed the disappearance of vascular markings in the entire right lung and upper left field, a severe mediastinal shift to the left, and a bilaterally flattened diaphragm. Chest CT revealed a giant emphysematous bulla occupying the right hemithorax, compressing almost the entire right lung parenchyma, and extending across the anterior mediastinum into the left hemithorax. Despite the administration of medication, his respiratory condition deteriorated further on Day 3 after hospitalization. Since the compression of the mediastinum by the expanding giant emphysematous bulla was thought to be the cause of the respiratory deterioration, a small-diameter tube was inserted percutaneously into the giant bulla for decompression. The intracavitary drainage relieved symptoms immediately and dramatically; however, a follow-up chest radiograph obtained 1 h after showed a mild right pneumothorax, requiring insertion of a chest tube into the right thoracic cavity to prevent further deterioration of the respiratory condition. His respiratory condition improved, but air leakage still persisted. CT after intracavitary drainage showed atelectasis of the right middle and lower lobes, which were expected to be reinflated by the removal of the giant bulla. Therefore, radical surgery was performed 9 days after the drainage. The giant bulla had herniated into the left hemithorax through the anterior mediastinum and was adhering to the left mediastinal pleura. Right upper lobectomy was successfully performed after dividing the fibrous adhesions and pulling the giant bulla into the right hemithorax. He was discharged on Day 13, remained asymptomatic, and had returned to normal daily activities by 10 months postoperatively.

**CONCLUSIONS:**

We conclude that intracavitary drainage can serve as an effective bridge to secure surgery in patients with giant emphysematous bullae causing the deterioration of respiratory condition.

## Abbreviation

mMRC
: modified Medical Research Council

## INTRODUCTION

Giant emphysematous bullae are known to gradually enlarge and compress adjacent lung lobes, resulting in a progressive impairment of respiratory function.^1)^ Intracavitary drainage is an option to relieve urgently deteriorating respiratory condition and may serve as a bridge to radical surgery^2)^ We herein report a rare case of a giant emphysematous bulla extending beyond the adjacent lobe to the contralateral side and causing respiratory failure due to severe mediastinal compression. It was successfully treated with emergency intracavitary drainage followed by elective right upper lobectomy.

## CASE PRESENTATION

A 57-year-old man presented to our department with progressive dyspnea extending over 1 month. He had a history of diabetes mellitus and bronchial asthma. His smoking history was 30 pack-years, and he had quit smoking 1 month prior to presentation. An emphysematous bulla had been noticed approximately 30 years earlier but had been left untreated. He was asymptomatic until 1 month before presentation, after which time dyspnea gradually worsened, leading to difficulty in ambulation. He was eventually transported to our emergency department.

On arrival, his systolic and diastolic blood pressures were 160 and 114 mmHg, respectively. He presented tachycardia with a heart rate of 110 beats/min. His respiratory condition deteriorated to a respiratory rate of 24 breaths/min and an oxygen saturation of 91% on 2 L/min of nasal oxygen. His Hugh–Jones classification and mMRC grade were class V and grade 4, respectively. Arterial blood gas analysis showed a pH of 7.416, partial oxygen pressure (PaO_2_) of 70 mmHg, and partial carbon dioxide pressure (PaCO_2_) of 48 mmHg on 2 L/min oxygen, respectively. Laboratory tests revealed a white blood cell count of 11690/μL and mild hyperglycemia (blood glucose level, 141 mg/dL; hemoglobin A1c, 7.5%), with no other significant abnormalities in biochemical or coagulation parameters. Chest radiographs demonstrated bilateral diaphragmatic flattening, disappearance of vascular markings throughout the entire right lung and the left upper lung field, and a significant leftward shift of the mediastinum and anterior junction line (**Fig. 1A**). Chest CT was performed in the left lateral decubitus position because the patient could not tolerate the supine position; it revealed a giant bulla occupying the right hemithorax and extending ventrally along the anterior mediastinum, crossing behind the sternum into the left hemithorax, and causing marked mediastinal compression (**Fig. 1B**).

**Fig. 1:**
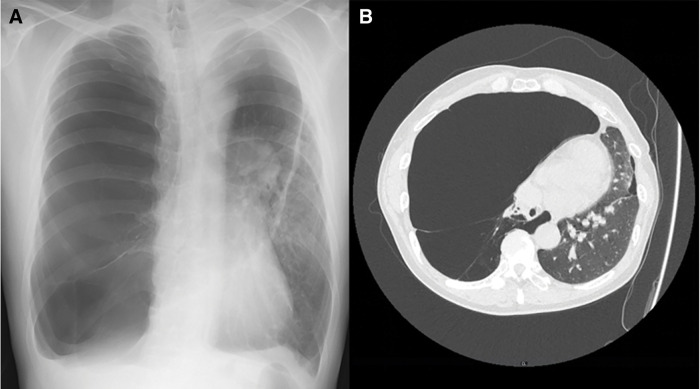
Radiological imaging at initial visit. (**A**) Chest radiographs showed a flattening of the bilateral diaphragms, loss of vascular markings throughout the right lung and the left upper lung fields, and leftward deviation of the mediastinum and an anterior junction line. (**B**) Chest plain CT radiograph, acquired in the left lateral decubitus position, showed a large cystic lesion extending from the entire right hemithorax toward the mediastinum and left hemithorax, and causing a mediastinal shift.

On admission, he required supplemental oxygen but was hemodynamically stable, and conservative medical management was initiated. On Day 3, his breathing became further labored and obviously worsened, and oxygen saturation dropped to 88%. A chest radiograph revealed a marked leftward mediastinal shift due to the expansion of the giant bulla. Emergency intracavitary drainage was performed using an aspiration kit (9 Fr, Argyle Fukuroi aspiration kit; Cardinal Health, Tokyo, Japan) (**Fig. 2A**), resulting in a rapid and dramatic improvement of dyspnea and stabilization of respiratory and circulatory status. As expected, the follow-up chest radiograph taken 1 h after the drainage revealed a mild right pneumothorax, which required the insertion of a chest tube into the right thoracic cavity (**Fig. 2B**).

**Fig. 2:**
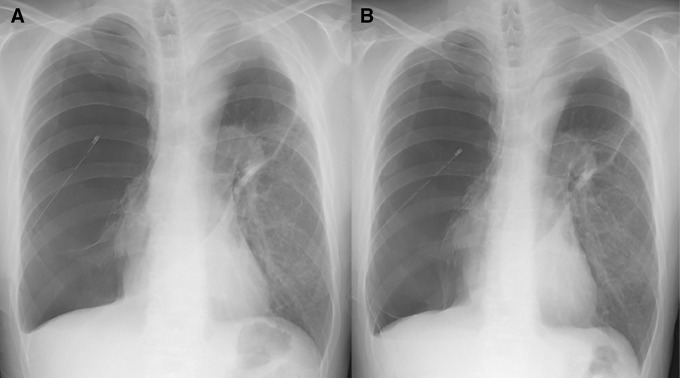
Chest radiographs acquired after drainage. (**A**) Intracavitary drainage with a small-diameter tube with a sharp tip. The flattening of the right diaphragm and the leftward mediastinal shift have been ameliorated. (**B**) The presence of a right pneumothorax was confirmed 1 h after intracavitary drainage.

After the procedures had been completed, the symptoms and respiratory function of the patient improved markedly (Hugh–Jones class IV to III, mMRC grade 4 to 3). Arterial blood gas analysis in room air showed an improvement in oxygenation (pH of 7.418, PaO_2_ of 64 mmHg, PaCO_2_ of 45 mmHg in room air). He became able to walk and engaged in respiratory rehabilitation. His 6-minute walk distance was 318 m in room air with a lowest oxygen saturation of 87% and a maximum heart rate of 130 beats/min. A chest radiograph showed a significant improvement in diaphragmatic flattening and mediastinal deviation, although a giant bulla and residual right pneumothorax persisted. Post-drainage chest CT still revealed a giant bulla occupying the right upper lobe and compressing the middle and lower lobes (**Fig. 3**). There appeared to be almost no normal lung parenchyma remaining in the right upper lobe. No obvious tumorous shadows were identified in the right lung. Pulmonary perfusion scintigraphy demonstrated no perfusion in the right upper lung field, whereas minimal perfusion was preserved in the lower lung field (**Fig. 4**). Air leakage continued from the chest tube upon expiration under water seal. Considering the persistent air leakage and the atelectasis of the right middle and lower lobes, radical surgical treatment was performed 9 days later to control the air leakage and to improve respiratory function through the re-expansion of the right middle and lower lobes.

**Fig. 3:**
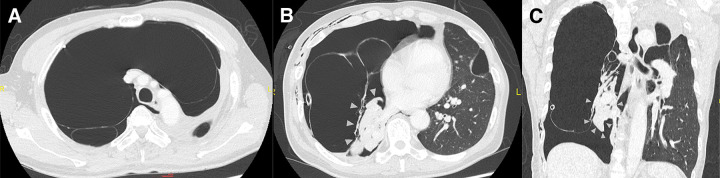
Chest plain CT after intracavitary drainage. (**A**) The giant bulla still extended into the contralateral hemithorax with adhesions at its apex. (**B**) The middle and lower lobes remained collapsed. (**C**) The coronal view showed the collapsed middle and lower lobes. Asterisk shows the right upper lobe bronchus. Gray triangles show the collapsed middle and lower lobes.

**Fig. 4:**
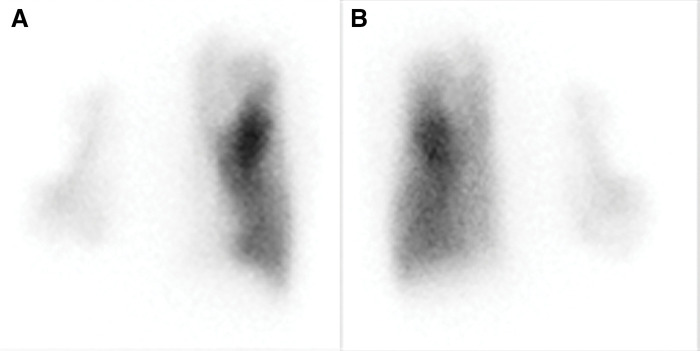
Pulmonary perfusion scintigraphy from the ventral (**A**) and dorsal (**B**) view. They illustrated no perfusion in the right upper lung field and minimal perfusion in the right lower and left upper lung field corresponding to the giant bulla.

A posterolateral thoracotomy was performed, anticipating limited visualization due to the giant bulla extending into the left hemithorax, and the need for manually extracting the giant bulla, which had been deeply adherent. The bulla originated predominantly from the right upper lobe and its tip extended into the left hemithorax through the anterior mediastinum behind the sternum, where it adhered broadly to the mediastinal pleura. The fibrous adhesions were cut up, and the giant bulla was pulled into the right hemithorax. It was observed by palpation that the lung parenchyma of the right upper lobe was nearly destroyed due to the giant bulla as expected from the findings of preoperative chest CT. We decided to perform a right upper lobectomy to resect the giant bulla. The fissures were divided using a stapler with reinforcement material. No air leakage was observed in the leakage test, and the staple line was further covered with a polyglycolic acid sheet and fibrin glue. The operating time was 195 min with an intraoperative blood loss of 15 g. Histopathological examination revealed no malignancy, confirming the diagnosis of an emphysematous bulla.

The patient required bronchoscopic aspiration because of sputum retention on POD 4 and 6. A postoperative persistent air leakage was observed, but the chest tube was removed on POD 9. The outcome of the 6-min walk test on POD 10 revealed a marked improvement compared with the result of the preoperative test (**Table 1**). The patient continued postoperative respiratory rehabilitation and was discharged on POD 13. Ten months after surgery, the patient remained asymptomatic and had returned to normal daily activity (**Fig. 5**).

**Table 1:** Comparison of the 6-min walk test results before and after surgery

6-min walk test	Preoperative	POD 10
Walk distance (m)	318	372
Minimum SpO_2_ (%) (room air)	87	94
Maximum pulse rate (/min)	130	116

SpO_2_, peripheral oxygen saturation

**Fig. 5:**
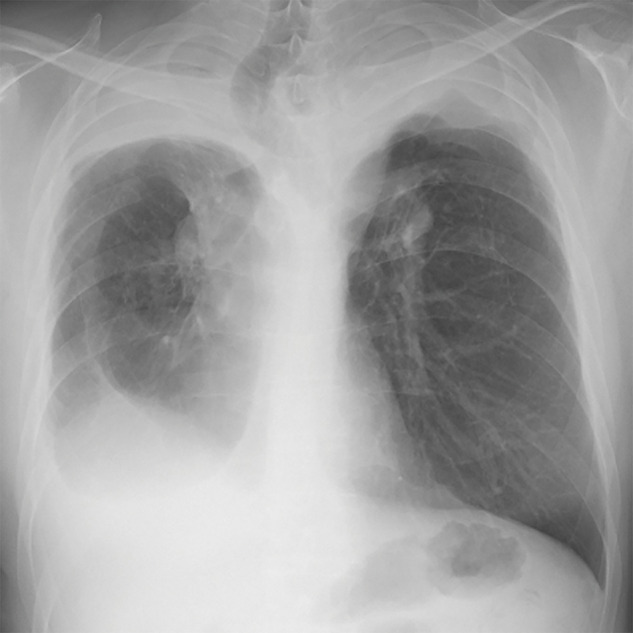
Chest radiograph acquired 10 months postoperatively. This revealed the absence of any mediastinal shift or flattening of the diaphragm.

## DISCUSSION

A giant emphysematous bulla is defined as a pulmonary cyst occupying more than one-third of a hemithorax and compressing adjacent lung parenchyma.^3)^ In our case, the bulla extended across the anterior mediastinum into the left hemithorax, causing severe mediastinal compression with resultant respiratory and circulatory instability. Percutaneous intracavitary drainage was therefore performed to relieve this obstructive shock condition, leading to rapid symptomatic improvement and saving his life. Decompression also enabled preoperative rehabilitation and detailed anatomical and functional assessment, allowing safe elective radical surgery to expect improved perfusion and ventilation of the right middle and lower lobes.

Macarthur and Fountain first reported symptomatic improvement in 28 of 31 patients with emphysematous bullae, for whom surgery was contraindicated, when they were treated with intracavitary drainage,^4)^ establishing this technique as a therapeutic option for giant emphysematous bullae. Subsequent reports have described various combined approaches, including surgical resection,^1)^ endobronchial occlusion using bronchial blockers,^5)^ and intrabullous instillation of sclerosing agents.^6)^ The benefits of intracavitary drainage include immediate decompression, improved respiratory function, and making it feasible to evaluate the re-expansion potential and structural reversibility of the compressed lung.^4)^ In the patient described here, drainage led to immediate symptomatic and physiological improvement, stabilizing the general condition and facilitating effective respiratory rehabilitation. It should be emphasized that the intracavitary drainage procedure serves as a bridge to definitive surgery or other treatments and as a means to prevent further deterioration of respiratory and circulatory function; therefore, it is not performed in all cases with emphysematous giant bullae. The indication of this procedure should be limited to patients whose cardiopulmonary stability cannot be maintained due to the expansion of a giant bulla. Regarding the procedure, a sharp-tipped, small-diameter tube is preferable for penetrating the thick wall of a giant bulla and preventing re-expansion edema caused by the immediate expansion of the compressed lung following rapid decompression of the giant bulla. A small-diameter tube may offer the advantage of preventing ventilatory failure caused by significant air leakage. Further, it should be anticipated that pneumothorax may occur after the intracavitary drainage.

We performed surgery because not only a giant emphysematous bulla with persistent air leakage remained but also the lung perfusion of the right lower field was barely maintained. Schipper et al. have reported that bullectomy for giant emphysematous bullae can be performed safely with acceptable morbidity and provides significant and durable improvement in respiratory function when patients are appropriately selected.^1)^ They further emphasized that favorable outcomes are strongly associated with preserved pulmonary perfusion and re-expansion capacity of the compressed lung, underscoring the importance of careful preoperative functional assessment. Consistent with these findings, our patient showed preserved pulmonary perfusion and re-expansion potential after intracavitary drainage, supporting elective surgical resection as the most definitive treatment. Preoperative functional improvement achieved by the intracavitary drainage allowed safer surgery with lung volume–reduction effects.

Finally, patients with emphysematous bullae have a higher incidence of lung cancer than the general population, which is probably related to smoking exposure, intrabullous carcinogen accumulation, and scar-associated carcinogenesis.^7)^ Reports of lung cancer diagnosed during surgery following intracavitary drainage further support this concern.^2)^ When the general condition of the patient permits it, surgical resection therefore represents a more comprehensive and appropriate treatment strategy compared with other conservative treatments.

## CONCLUSIONS

We encountered a case of a giant emphysematous bulla causing respiratory failure due to mediastinal compression that was successfully treated with percutaneous intracavitary drainage followed by elective right upper lobectomy. Intracavitary drainage using a small-diameter tube with a sharp tip can serve as an effective bridge to secure radical surgery.

## DECLARATIONS

### Availability of data and materials

There is no dataset supporting the conclusions of this article.
